# mtDNA in the Pathogenesis of Cardiovascular Diseases

**DOI:** 10.1155/2021/7157109

**Published:** 2021-11-09

**Authors:** Lili Wang, Qianhui Zhang, Kexin Yuan, Jing Yuan

**Affiliations:** Department of Cardiology, Hebei General Hospital, Shijiazhuang, Hebei Province, China

## Abstract

The incidence rate of cardiovascular disease (CVD) has been increasing year by year and has become the main cause for the increase of mortality. Mitochondrial DNA (mtDNA) plays a crucial role in the pathogenesis of CVD, especially in heart failure and ischemic heart diseases. With the deepening of research, more and more evidence showed that mtDNA is related to the occurrence and development of CVD. Current studies mainly focus on how mtDNA copy number, an indirect biomarker of mitochondrial function, contributes to CVD and its underlying mechanisms including mtDNA autophagy, the effect of mtDNA on cardiac inflammation, and related metabolic functions. However, no relevant studies have been conducted yet. In this paper, we combed the current research status of the mechanism related to the influence of mtDNA on the occurrence, development, and prognosis of CVD, so as to find whether these mechanisms have something in common, or is there a correlation between each mechanism for the development of CVD?

## 1. Introduction of mtDNA Structure and Function

[1] mtDNA itself is a small, double-stranded circular molecule, and human mtDNA is 16,569 bp in length. mtDNA encodes 13 polypeptide subunits of the oxidative phosphorylation (OXPHOS) enzyme complexes, 22 transfer RNAs, and 2 ribosome RNAs, which are needed for mitochondrial respiration [2]. Unlike nuclear DNA (nDNA), mtDNA lacks histone protection and efficient DNA repair function, making it vulnerable to damage. We know that DNA is the major genetic material and contains genetic information. About 30% of the genetic profile in mtDNA is used to encode, and about 70% is used to encode the proteins within the mitochondria, so the disease caused by changes in mtDNA will have the corresponding genetic nature. A growing number of studies have shown that mtDNA plays an important role in the regulation of innate immunity and can induce inflammatory disease via affecting cell stress. Mitochondrial dysfunction, resulting in release of mtDNA called damage-associated molecular pattern (DAMP), can trigger natural immunity, leading to DAMP activation of inflammation. Furthermore, the characteristics of mtDNA and bacterial DNA are similar; 30 released mtDNA molecules can cause CVDs such as atherosclerosis by inducing inflammatory reaction. The decrease of mtDNA increased autophagy regulating function, and CVD after myocardial cell damage can also lead to line body dysfunction, release more mtDNA to cells, and cause CVD through various mechanisms, which form a circle. Therefore, it is significant to study the multiple mechanisms of mtDNA on the occurrence, development, and prognosis of CVD, which can provide new ideas for clinical treatment.

## 2. mtDNA Copy Number and CVD

mtDNA is the only relatively independent genome that exists in organelles. It can control and encode part of proteins, and it can indirectly reflect mitochondrial function. The copy number of mtDNA in the genome can predict CVD's occurrence, development, and prognosis. Foram et al. showed that the copy number of mtDNA is inversely proportional to CVD events, and the influence on CVD risk assessment of young individuals is significantly increased [3]. However, the sample size of the above studies is small. Therefore, multi-factor analysis of age, region, sex, blood lipid, blood pressure, smoking status and related drugs should be studied and to determine the application potential of mtDNA in clinic. Christina et al. evaluated 2,507 African-Americans and European-Americans for community arteriosclerosis risk and studied the relationship between mtDNA-CN and nDNA methylation. They have shown that the regulation of mtDNA copy number leads to differences in the methylation and expression of genes associated with signal transduction processes, thus affecting the expression status of crucial genes in human pathophysiologic process [4]. In addition, mtDNA copy number plays a certain role in the development of CVD. mtDNA copy number may reflect the degree of mtDNA damage and may be a biomarker of mitochondrial function and a predictor of cardiovascular disease risk. It has been reported in the literature that mtDNA copy number in the cardiomyocytes of patients with heart failure is significantly decreased. Oxidative stress can aggravate heart failure and cardiac remodeling, and the electron transport chain in mitochondria is an important source of oxidative free radicals. In myocardial infarction mice, the expression of the antioxidant gene peroxiredoxin -3 (PRX-3) and mtTFA can, to some extent, increase mtDNA copy number in patients with heart failure, delay pathological myocardial remodeling, and improve the survival rate of mice. Ikeda et al. found that in mice overexpressing mtTFA and Twinkle helical protein, mtDNA copy number was increased by 2 times and myocardial damage in isolated cardiac hypertrophy was alleviated [5]. Therefore, increasing mtDNA copy number becomes a new way to treat myocardial injury mediated by oxidative stress.

mtDNA copy number variation is influenced by environmental and genetic factors, among them, oxidative stress is one of the most important factors. Different effects of these factors on mtDNA copy number need more research to clarify. At the same time, how mtDNA copy number variation works on the prediction of the occurrence and development of diseases and its appropriate application in treatment also will be the important direction of research in the future.

## 3. mtDNA Inflammatory Response

“Aseptic inflammation of the heart” is usually a secondary response to myocardial damage caused by ischemia injury or heart failure. A growing number of researches have demonstrated that inflammation plays an important role in the physiological and pathological progress of heart failure [6]. The expression level of inflammatory factors in patients with heart failure was significantly higher than that in healthy people [7, 8]. The abnormal expression of inflammatory factors and their receptors are closely associated with the mortality of patients with severe heart failure and the poor prognosis of idiopathic dilated cardiomyopathy [9–11]. Besides, studies have identified that the expression status of TNF-*α* in serum is closely related to the severity of heart failure [7, 8]. Relative hypomethylation, unique structural characteristics, and vulnerability to oxidative damage make mtDNA a potentially potent DAMP in disease, which activates innate immunity to induce inflammation and type I interferon responses. Recent studies have found that pattern recognition receptors (PRRs), including TLR-9, NLRP3 inflammasome, and GMP-AMP synthase- (cGAS-) STING pathway, are key promoters of mtDNA-related inflammatory responses [12, 13]. The proinflammatory function of mtDNA was first identified in 2004 by Collins et al. [14]. In this study, the researchers injected mtDNA instead of nDNA into the articular cavity of mice and found that mtDNA could promote the inflammatory response of joints by inducing the secretion of TNF-*α* in spleen cells. At the same time, it has been reported that loss of mitochondrial DNA attenuates IL-1*β* secretion in macrophages by inhibiting activation of inflammasome after treatment with lipopolysaccharide (LPS) and ATP [15]. The way mtDNA participates in inflammatory response is similar to that of bacteria. Significantly, oxidation is necessary in mtDNA-related inflammatory response [16, 17]. When mtDNA binds to TFAM, nucleotide has a high stability; otherwise, mtDNA is in a fragile and easily degraded state. It has been reported that the abnormal expression of TFMA or mtDNA caused by oxidative modification is the decisive factor leading to the combination of TFAM and mtDNA and the instability of nucleoid. However, the role of defective TFAM and mtDNA binding in inflammatory response is still unknown. The study found showed that both cell-free mtDNA and TFAM-bound mtDNA play an important role in inflammatory response [18].

### 3.1. mtDNA, as an Inflammatory Mediator, Interacts with the following Substances to Produce an Autoimmune Stress Response and Trigger Inflammation

#### 3.1.1. Toll-Like Receptor 9

Studies had shown that acute myocardial infarction can cause aseptic inflammation, aggravate tissue damage, and lead to increased levels of mtDNA, which activates the NF-*κ*B pathway through TLR9 and triggers an innate immune response, leading to myocardial cell damage. In arteriosclerosis, the released mtDNA forms a complex with the human antimicrobial peptide LL-37, which is resistant to degradation of deoxyribonuclease 2 and escapes autophagy recognition, leading to the activation of TLR9-mediated inflammatory response and the sustained activation of TLR9, causes autoimmune activation of chemokines and live cytokines, and exacerbates arteriosclerosis [19]. Increased mtDNA in plasma source exosomes in chronic heart failure triggers an inflammatory response through the TLR9-NF-*κ*B pathway [20]. mtDNA released in acute myocardial infarction activates TLR9 and aggravates ischemia reperfusion injury through the TLR9-p38 MAPK pathway, thus exacerbating myocardial injury. Myocardial ischemia-reperfusion injury is a challenging clinical problem. During myocardial ischemia-reperfusion injury, a large amount of mtDNA is released, which causes inflammatory reaction and aggravates myocardial injury [21]. Current studies suggest that chloroquine interferes with TLR9-mediated inflammatory signaling and transduction, and PDTC inhibits NF-*κ*B and inhibits NF-*κ*B activation by metal chelation. More suitable drugs and dosages require further study [22].

#### 3.1.2. NLRP3 and Inflammasomes

NLRP3 is the second factor that has been shown to influence the inflammatory response through REDOX and mtDNA. NLRP3 is very sensitive to bacterial infection, viral invasion, and other pathogenic factors [23, 24]. It was found that mtROS activated NLRP3, and the mechanism was closely related to the oxidation of mtDNA [15, 16, 25, 26]. mtROS can influence mtDNA subcellular localization and activate NLRP3 inflammasome by binding NLRP3 [16]. NLRP3 inflammasome is a multiprotein complex composed of NLRP3, which is a spot-like protein with a caspase activation and recruitment domain and is closely related to apoptosis. It has been reported that NLRP3 and ASC colocate to mitochondrial clusters in the endoplasmic reticulum-perinuclear space when NLRP3 inflammasome is activated, thereby inducing caspase 1 cleavage and activation [27]. When caspase-1 is activated, the expression levels of proinflammatory factors such as IL-1 and IL-18 are significantly increased and thus participate in REDOX inflammatory reactions [28]. Besides, deletion of NLRP3 and caspase-1 genes reduces mtDNA release [15, 26]. It has also been found that nonoxidative mitochondrial DNA can induce IL-1*β* secretion by activating inflammatory bodies such as AIM2 [29]. Multiple risk factors of CVD are also considered to activate inflammasomes, so intervention in the activation of inflammasomes can be a prevention and treatment target of cardiovascular disease. Heart function of I/R mice with NLRP3 defect was improved, and hypoxia injury was reduced. Under the action of ATP, ischemic myocardial fibroblasts promote the assembly and upregulation of NLRP3 inflammasomes, thus affecting the infarct size. By constructing a mouse model of ischemic injury, the use of NLRP3 inflammasome inhibitors prevented myocardial cell death and reduced left ventricular systolic function in mice to a certain extent. In heart failure following ischemic heart injury, a strong inflammatory response leads to further injury and dysfunction of cardiac contraction and expansion, and the release of cell debris during tissue injury causes conformational changes in NLRP3 inflammasomes, leading to the activation of caspase-1.Clinical treatment of heart failure patients with IL-1 antagonist injection showed increased oxygen consumption, which is expected to be a new direction of heart failure treatment in the future.

Currently, the NLRP3 inflammasome's influence on heart failure has been largely focused on its mediated role in IL-1 and IL-18 in heart remodeling. The activation of NLRP3 inflammasomes in mice had been shown to induce heart failure by promoting myocardial inflammation and systolic dysfunction by producing proinflammatory IL-1 [30].

#### 3.1.3. Cyclic GMP–AMP Synthetase

When first discovered, the CGAS-STING signaling pathway was thought to be a crucial component of the innate immune system; its function being to detect the existence of cytoplasmic DNA and induce the secretion of inflammatory factors. When the cyclic GMP-AMP synthase (cGAS) senses DNA that should not be present in the cytoplasm, it catalyzes a chemical reaction between GTP and ATP that produces a small molecule called cyclic GMP-AMP (cGAMP); the molecule acts as a messenger for innate immune system, further activating the immune response. Therefore, cGAS is like a burglar alarm, and cGAMP is the electrical signal generated by the burglar alarm [31, 32]. In recent years, STING has been identified as a cytoplasmic anchored protein capable of directly binding to double-stranded DNA or initiating IFN response via cyclic dinucleotide activation. In addition, the IFN response initiated by STING can also be realized by affecting mtDNA [33, 34]. The mechanism is that when STING binds to mtDNA, cGAS will begin to recruit STING proteins, and STING induces phosphorylation of transcription factor IRF-3 through activation of TANK (NF-*κ*B activator associated with TRAF family members) binding kinase and NF-*κ*B signaling pathway [35]. mtDNA also activates cGAS-STING to initiate the production of type 1 interferon (IFNS), which triggers an inflammatory response [36]. Recent literature suggests that mtDNA released into the cytoplasm during apoptosis may activate cGAS to produce CGAMP, which enlists STING, and downstream to further activate TBK1-IRF3 signaling pathway to produce IFN-7 and IFN-8. In addition, recent studies have shown that mtDNA produced by herpes virus infection can also activate the cGAS-STING signaling pathway 9. Activated IRF-3 mediates transcription of nuclear gene products stimulated by type I and type III IFN and IFN leading to mtDNA-induced inflammatory response. In physiological environment, oxidative mtDNA whole-body injection increased IFN-stimulated gene expression in the spleen of wild-type mice, but not in STING-deficient mice. Therefore, the study of CGAS and STING targeted drugs for the treatment of chronic inflammation has a good prospect.

## 4. mtDNA Autophagy and Cardiovascular Disease

The inflammatory response triggered by mtDNA is closely related to autophagy. Autophagy is a key adaptive mechanism by which excess cytoplasmic components (such as DNA, proteins, mitochondria, and intracellular pathogens) are “confiscated” by double-membrane vacuoles (autophages) that fuse with lysosomes to degrade the cytoplasmic components captured in them for clearance, to protect the cells from stress-induced damage [37]. The pathological process of a variety of CVD is accompanied by changes in the autophagy of cardiac myocytes. Myocardial injury activates autophagy's repair of cells. If the damage is severe, it could cause apoptotic process. In addition, autophagy can limit the inflammatory response by regulating key immune regulators such as NF-*κ*B10 and STING11. In pathological conditions such as microbial infection and major trauma, when malfunctioning mitochondria and damaged mtDNA accumulate in the cytoplasm beyond the capacity of autophagy, the negative regulatory function of autophagy is lost. As a result, the inflammatory response increased significantly, often accompanied by chronic inflammation, which is caused by abnormal responses to TLR92, TLR12, NLRP35, and/or cGAS-STING13, thus triggering the occurrence and development of CVD [38].

Therefore, the research on autophagy inducers will help to improve the loss of autophagy caused by mtDNA, thus reducing the impact of chronic inflammation on CVD. mtDNA that escapes autophagy can trigger an inflammatory response in cardiomyocytes mediated by Toll-like receptor TLR9 and can induce myocarditis and dilated cardiomyopathy [39].

This study provided a new idea for elucidating the mechanism of chronic inflammation in heart failure. By forming a complex with human antimicrobial Peptide LL-37, mtDNA from dead cells or from the structure of NET not only can not be eliminated by DNASE II, the LL-37-mtDNA complex formed at the same time, after entering into the cells, becomes difficult to clear by avoiding the autophagy recognition, resulting in the continuous activation of the TLR9 signaling pathway in the cells [40].

## 5. mtDNA Mutations and Cardiovascular Disease

In view of the structure of mtDNA and its own genetic characteristics, mtDNA is prone to mutation. Holt and Wallace initially detected mtDNA mutations, respectively, in cells from patients with mitochondrial encephalomyopathy and Leber hereditary neuropathy. Mutations are possible candidate risk factors for cardiovascular disease due to the effect of mitochondria on energy metabolism and the generation of reactive oxygen species by evidence-based complementary and alternative drugs. With the improvement of scientific research level, multiple researches have demonstrated that CVD and mtDNA mutations are closely related, such as heart block, myocarditis, and sudden death. These studies provided theoretical and experimental basis for elucidating the pathogenesis of CVD. However, the current research is limited to the detection of some mtDNA mutations in some CVD, but how do the mutated genes change the structure and function of the proteins they encode and how the mtDNA directed proteins affect the occurrence and development of mitochondrial diseases caused by oxidative stress and energy metabolism remain to be further studied.

## 6. mtDNA and Metabolic Memory in Diabetic Cardiomyopathy

More than 40 years ago, some scholars discovered a disease that can occur independently of cardiovascular disease or hypertension, which was named diabetic cardiomyopathy (DCM). At present, its pathogenesis has not been fully clarified [41]. Multiple pathophysiological mechanisms of DCM had been identified, such as the existence of hyperglycemia, nonenzymatic glycosylation of macromolecules (such as proteins), disorder of energy metabolism, mitochondrial dysfunction, improper calcium treatment, abnormal reactive oxygen species, inflammation, cardiomyocyte death, and cardiac hypertrophy and fibrosis, resulting in impaired cardiac systolic function. There was also growing evidence that a phenomenon called “metabolic memory” exists in cardiovascular complications of diabetes, suggesting that these pathogenic mechanisms might be controlled by mtDNA epigenetic modifications. While how epigenetic mechanisms work is still not fully understood, a series of studies have demonstrated its importance in modulating the cardiac response to diabetes, and these studies are believed to contribute to an in-depth comprehending of the specific mechanisms of DCM and their possible prevention and/or treatment. Cong et al. found that mitochondrial glucose and lipid metabolism disorders were one of the important mechanisms of DCM. There were two ways to increase ROS in myocardium: mitochondrial and extramitochondrial [42, 43]. Boudina et al. study found enhanced mitochondrial uncoupling and mitochondrial respiratory chain dysfunction in cardiomyocytes of DB/DB mice with type 2 diabetes [44]. In addition, increased UCP3 expression, mitochondrial damage, and reduced heart energy efficiency were reported after cardiac ischemia in DB/DB mice [45]. The dysregulation of mitochondrial Ca2+ in DCM is a research hotpot in these years, and the presence of mitochondrial Ca2+ regulation disorders in diabetic animal models had been reported in laboratory [46, 47]. Mitochondrial dysfunction has a significant effect on the occurrence and progression of diabetic cardiomyopathy. mtDNA damage of myocardial cells caused by oxidative stress affects the pathogenesis of diabetic cardiomyopathy significantly. Mitochondrial oxidative stress was considered to be the single cause of mtDNA damage and the underlying cause of mitochondrial dysfunction. The formation of 8-hydroxy-2′-deoxyguanosine (8-OHDG) is a classic manifestation of mtDNA damage, which leads to mutations when the 8-oxyguanosine DNA glycosylase (Ogg1) is not repaired properly; Cividini et al. showed that high glucose induced o-GlcNAcylation of Ogg1 and increased mtDNA damage [48]. Hicks et al. showed that increased oxidative stress induced by diabetes would change the function of mitochondrial topoisomerase, and the activation/inhibition state of mitochondrial topoisomerase would have an important impact on mtDNA integrity and myocardial health in diabetes mellitus. Although mtDNA repair mechanisms have been demonstrated in cardiomyocytes, the mechanisms of this repair have not been fully identified [49]. Therefore, these findings provided a new possible biochemical mechanism for diabetic cardiomyopathy.

## 7. Summary

**Figure 1:**
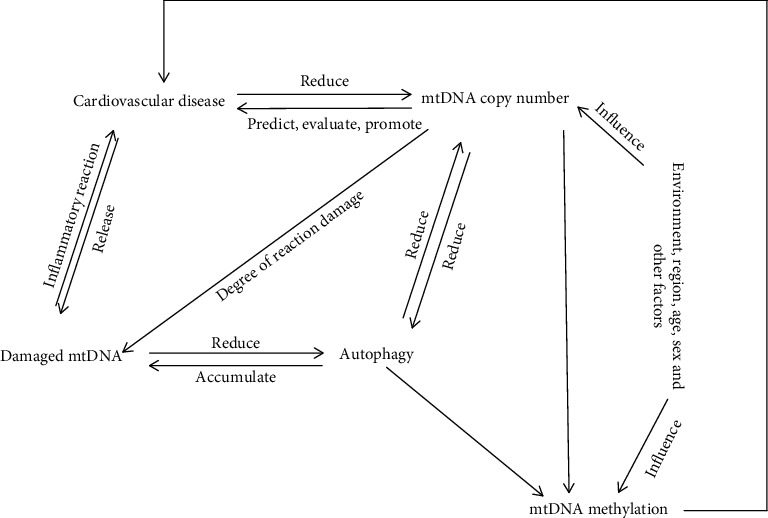
Relationship between mtDNA and cardiovascular disease. Multiple mechanisms interact to influence the occurrence, development, and prognosis of cardiovascular disease.

From the literature, it is clear that mtDNA plays an important role in keeping mitochondria normal (Table 1). The copy number of mtDNA can reflect the occurrence and development of CVD to some extent. mtDNA damage not only causes a variety of related metabolic diseases in human body but also is an important point of CVD and causes of new coronary CVD. mtDNA is special due to its composition and structure, such as no histone protection and located near the respiratory chain, the main site of endogenous oxygen radicals. It is vulnerable to damage by free radicals and is not easy to repair due to its poor damage repair ability. Because mtDNA is directly related to energy metabolism in vivo, mtDNA damage can easily lead to reduced ATP synthesis and cause cell senescence and even death. Apoptosis or necrosis is regulated by multiple factors, among which mtDNA damage may be one of the effects of many mechanisms on cardiovascular disease. The physiological mechanism network system constructed by mtDNA copy, injury, senility, apoptosis, autophagy, and so on plays an important role in the occurrence of CVD (Figure 1). The combined effect of mtDNA on CVD needs to be further studied, and the effects of mtDNA polymorphism on cardiovascular diseases should also be studied.

## Figures and Tables

**Table 1:** Cardiovascular diseases associated with pathogenic mtDNA mutations.

Mutation type	Mutant gene mutations in the genes	Disease	Reference
Basepair substitution	mtDNA A4401G	Hypertension	[50]
	mtDNA T14484G		[51]
tRNA genetic mutation	tRNA^Met^ m.A4435G tRNA^Leu^ m.A3243G tRNA^Leu^ m.A4263G tRNA^Leu^ m.T4353C tRNA^Phe^ m.C593T tRNA^Trp^ m.C5553T tRNA^Glc^ m.T4353C tRNA^Ile^ m.T4291C tRNA^Leu^ m.T3253C tRNA^Ala^ m.A5655G		[52–58]
mRNA mutation	m.A8701G in ATP6		[59]
tRNA genetic mutation	tRNA^Thr^ m.G15927A tRNA^Phe^ m.T5592C tRNA^Leu^ m.A3243G	Coronary atherosclerotic disease	[60]
mtDNA deletion	mtDNA4977bp Deletion		[61]
Basepair substitution	mtDNA T16189C		[62]
mtDNA deletion	mtDNA4977bp Deletion	Arrhythmia	[63]
Basepair substitution	mtDNA T14709C	Cardiomyopathy	[64]
tRNA genetic mutation	tRNA^Alle^ m.4322dupC		[65]
	tRNA^Leu^ m.A3243G		[66]
mtDNA rearrangement deletion	7.5 KB deletion between ATPASE6 gene and D loop		[67]
	mtDNA7436bp Deletion		[67]
tRNA genetic mutation	tRNA^Leu^m.A3243G	Heart failure	[68]

## Data Availability

No data were used to support this study.
